# The Binary Toxin CDT of *Clostridium difficile* as a Tool for Intracellular Delivery of Bacterial Glucosyltransferase Domains

**DOI:** 10.3390/toxins10060225

**Published:** 2018-06-01

**Authors:** Lara-Antonia Beer, Helma Tatge, Carmen Schneider, Maximilian Ruschig, Michael Hust, Jessica Barton, Stefan Thiemann, Viola Fühner, Giulio Russo, Ralf Gerhard

**Affiliations:** 1 Institute of Toxicology, Hannover Medical School, 30625 Hannover, Germany; beer.lara-antonia@mh-hannover.de (L.-A.B.); tatge.helma@mh-hannover.de (H.T.); schneider.carmen@mh-hannover.de (C.S.); barton.jessica@mh-hannover.de (J.B.); thiemann.stefan@mh-hannover.de (S.T.); 2 Department of Biochemistry and Biotechnology, Technical University Braunschweig, 38106 Braunschweig, Germany; m.ruschig@tu-braunschweig.de (M.R.); m.hust@tu-braunschweig.de (M.H.); v.fuehner@tu-braunschweig.de (V.F.)

**Keywords:** binary toxins, *Clostridium difficile*, ADP-ribosyltransferase, glucosyltransferase, protein delivery

## Abstract

Binary toxins are produced by several pathogenic bacteria. Examples are the C2 toxin from *Clostridium botulinum*, the iota toxin from *Clostridium perfringens,* and the CDT from *Clostridium difficile*. All these binary toxins have ADP-ribosyltransferases (ADPRT) as their enzymatically active component that modify monomeric actin in their target cells. The binary C2 toxin was intensively described as a tool for intracellular delivery of allogenic ADPRTs. Here, we firstly describe the binary toxin CDT from *C. difficile* as an effective tool for heterologous intracellular delivery. Even 60 kDa glucosyltransferase domains of large clostridial glucosyltransferases can be delivered into cells. The glucosyltransferase domains of five tested large clostridial glucosyltransferases were successfully introduced into cells as chimeric fusions to the CDTa adapter domain (CDTaN). Cell uptake was demonstrated by the analysis of cell morphology, cytoskeleton staining, and intracellular substrate glucosylation. The fusion toxins were functional only when the adapter domain of CDTa was *N*-terminally located, according to its native orientation. Thus, like other binary toxins, the CDTaN/b system can be used for standardized delivery systems not only for bacterial ADPRTs but also for a variety of bacterial glucosyltransferase domains.

## 1. Introduction

*Clostridioides difficile* is a nosocomial pathogen that causes antibiotic-associated infections with mild to severe diarrhea, pseudomembranous colitis, toxic megacolon, or death, depending on the severity of the infection [1]. 

Pathogenic strains produce two main pathogenicity factors, i.e., toxin A (TcdA) and B (TcdB), which glucosylate Rho GTPases within the host cell cytosol [2]. Besides these single-chain AB toxins, some *C. difficile* strains produce a binary AB toxin called CDT [3]. In a 2008 survey, approximately 23% of 389 *C. difficile* isolates from 34 European countries tested positive for CDT [4]. In the last few decades, this binary toxin has come into focus as it occurs in the so-called hypervirulent *C. difficile* strains associated with higher morbidity and mortality [5,6,7].

Together with *Clostridium botulinum* C2 toxin, *Clostridium perfringens* iota toxin, *Clostridium spiroforme* toxin (CST), and *Bacillus cereus/thuringiensis* vegetative insecticidal proteins (VIP), CDT belongs to the family of binary ADP-ribosylating toxins. These binary toxins consist of two separate components: an enzymatically active ADP-ribosyltransferase (ADPRT) fused to an adapter and a binding and translocation component. Because of high sequence identity and structural similarities between iota toxin, CST, and CDT, these toxins are further classified as iota-like binary toxins [8,9]. 

CDTa contains an *N*-terminal signal sequence which is cleaved by proteolysis leaving an active 48 kDa protein [10]. Crystal structure analysis revealed that this protein has a two-domain structure, with the *C*-terminal portion being the ADPRT, and the *N*-terminal portion necessary for binding to CDTb [11]. Structure–function studies with iota toxin and *Bacillus anthracis* protective antigen, which shares 36% sequence identity with CDTb, provided information about CDTb domain structure that resulted to consist of an *N*-terminal activation domain I followed by domain II, necessary for membrane insertion and pore formation, domain III, which is involved in oligomerization, and the *C*-terminal domain IV, providing receptor binding [9]. The latter domain can be exchanged among CDTb, Ib (iota toxin binding component), and CSTb (CST binding component) without affecting the delivery of the enzymatic component (CDTa, Ia, or CSTa) [8], as they share the same receptor on host cells, namely, the lipolysis stimulated lipoprotein receptor (LSR) [12,13]. In the proposed model of uptake, the 99 kDa protein CDTb is activated by a serine protease, leaving an active 75 kDa fragment which binds to LSR and oligomerizes into heptamers. Whether oligomerization occurs before or after binding to the receptor is still unclear. However, after binding of CDTa, the CDTa/b complex is taken up into cells via receptor-mediated endocytosis. A decrease of the endosomal pH induces membrane insertion and pore formation by the CDTb oligomers, followed by translocation of CDTa into the cytosol. Inside the cytosol, CDTa ADP-ribosylates monomeric actin, leading to depolymerization of actin filaments and, therefore, breakdown of the actin cytoskeleton [14].

The delivery of the enzymatic component into the host cell cytosol by binary toxins is a highly complex but efficient process. In recent years, researchers have exploited this system as a molecular tool for the transport of different cargo into cells [15]. The *B. anthracis* binary toxin was one of the first binary delivery systems used for the transport of heterologous proteins or DNA. For example, fusion of the protein of interest to the lethal factor (LF) provides efficient uptake of this protein into target cells when applied with protective antigen (PA), the binding component of *B. anthracis* binary toxin [16]. To date, the PA/LF system is the only one that has been used for delivery of a glucosyltransferase domain [17]. Binary toxins belonging to the family of ADP-ribosylating binary toxins are as well useful transport systems for the uptake of fusion proteins. The C2 toxin was extensively studied for the delivery of, e.g., C3 toxin from *Clostridium limosum* [18] or *Staphylococcus aureus* [19] as a C2–C3 fusion protein, *Salmonella enterica* virulence factor SpvB as a C2IN–C/SpvB fusion protein [20], biotinylated proteins bound to a C2IN–streptavidin fusion protein [21], or p53 as a C2IN–p53 fusion protein [22] into the cytosol. Besides C2 from *C. botulinum*, the iota toxin is also suitable as a transport system for C3 [23]. 

To date, CDT has not yet been described as a transporter system. Here, we show that this system is effective in introducing fusion proteins into cells and is therefore an alternative to other binary toxin systems. We show that CDTb is eligible to transport large CDTaN fusion proteins, like the glucosyltransferases from *C. difficile* TcdA and TcdB, *Clostridium sordellii* lethal toxin (LT) and hemorrhagic toxin (HT), and *Clostridium novyi* α-toxin, into the cytosol, maintaining their functionality, as we confirmed with appropriate assays.

## 2. Results

### 2.1. Cloning and Characterization of CDT

**Figure 1:**
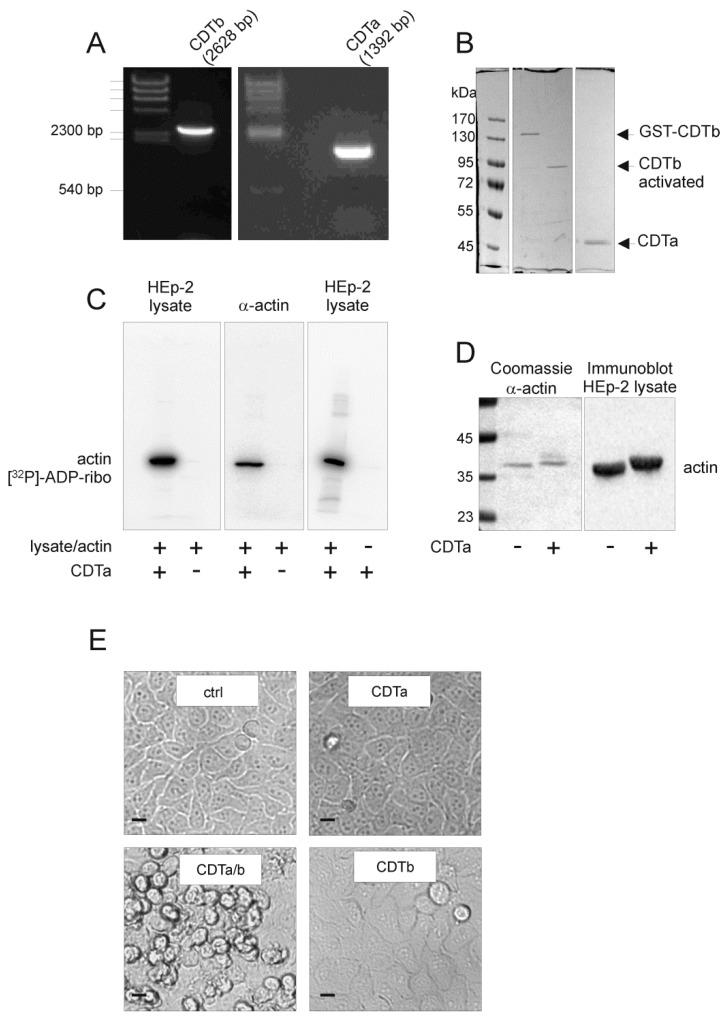
Cloning and functional characterization of recombinant CDT. (**A**) The open reading frames (ORF) of *cdta* (1392 bp) and *cdtb* (2631 bp) were amplified from genomic DNA of *Clostridium difficile* strain R20291; (**B**) The mature proteins CDTa (aa 43–463) and CDTb (aa 43–876) were expressed as GST fusion proteins. Whereas CDTa (48 kDa) was cleaved by thrombin from the GST tag, GST–CDTb was directly incubated with trypsin, resulting in the activated CDTb (75 kDa), as shown in Coomassie-stained SDS-PAGE; (**C**) The in vitro ADP-ribosyltransferase activity of CDTa was tested in a [^32^P]ADP-ribosylation assay. Filmless autoradiography shows in vitro [^32^P]ADP-ribosylated α-actin of HEp-2 cell lysates (left panel) and α-actin from rabbit muscle (middle panel). The right panel shows no auto-ADP-ribosylation of CDTa; (**D**) A gel shift assay of rabbit muscle α-actin (Coomassie staining) and α-actin from lysates of CDT-treated HEp-2 cells (immunoblot from whole cell assay) shows different apparent migration of ADP-ribosylated actin in SDS-PAGE; (**E**) Cell culture assay proving that CDTa/b was functional. HEp-2 cells were incubated with the subunit CDTb (1 µg/mL), the subunit CDTa (3 µg/mL), or the combination of CDTa and CDTb at their respective concentrations. Only combined CDTa and CDTb induced morphological changes compared to the untreated control cells. Scale bars represent 10 µm.

The complete open reading frame of *cdta* and *cdtb* were amplified by PCR from genomic DNA of *C. difficile* clade 2 hypervirulent strain R20291. The 1392 bp (CDTa) and 2631 bp (CDTb) (Figure 1A) fragments were ligated into pGEX-2T vector and were used for subsequent cloning of the mature proteins lacking their first 42-amino acid (aa) leader sequence for export. Mature CDTa (aa 43–63) and CDTb (aa 43–876) were expressed as GST fusion proteins. Whereas mature CDTa was released from GST by thrombin, GST–CDTb was directly digested by trypsin to result in activated CDTb (Figure 1B). In the following, CDTa and CDTb always stand for mature CDTa and activated CDTb, respectively, if not indicated otherwise. The enzymatic activity of CDTa was tested in ADP-ribosylation assays. The filmless autoradiography showed [^32^P]ADP-ribosylation of 42 kDa α-actin in HEp-2 cell lysates (Figure 1C). Purified α-actin from rabbit muscle was also [^32^P]ADP-ribosylated. There was no evidence of auto-[^32^P]ADP-ribosylation of 48 kDa CDTa (Figure 1C, right panel), as it is known from other classes of bacterial ADP-ribosyltransferases [24]. ADP-ribosylation of α-actin from HEp-2 cell lysates and purified α-actin was also tested in a gel shift assay where ADP-ribosylation of actin leads to a higher apparent molecular mass (Figure 1D). After characterization of the ADP-ribosyltransferase activity of CDTa, we tested functional CDTb in combination with CDTa in cell culture assays. The combination of CDTa and CDTb induced typical cell rounding as a consequence of the destruction of the actin cytoskeleton after 5 hours (Figure 1E). Neither CDTa nor CDTb alone induced any morphological effects on HEp-2 cells. 

### 2.2. C-Terminal Fusion Proteins are Delivered into Target Cells

**Figure 2:**
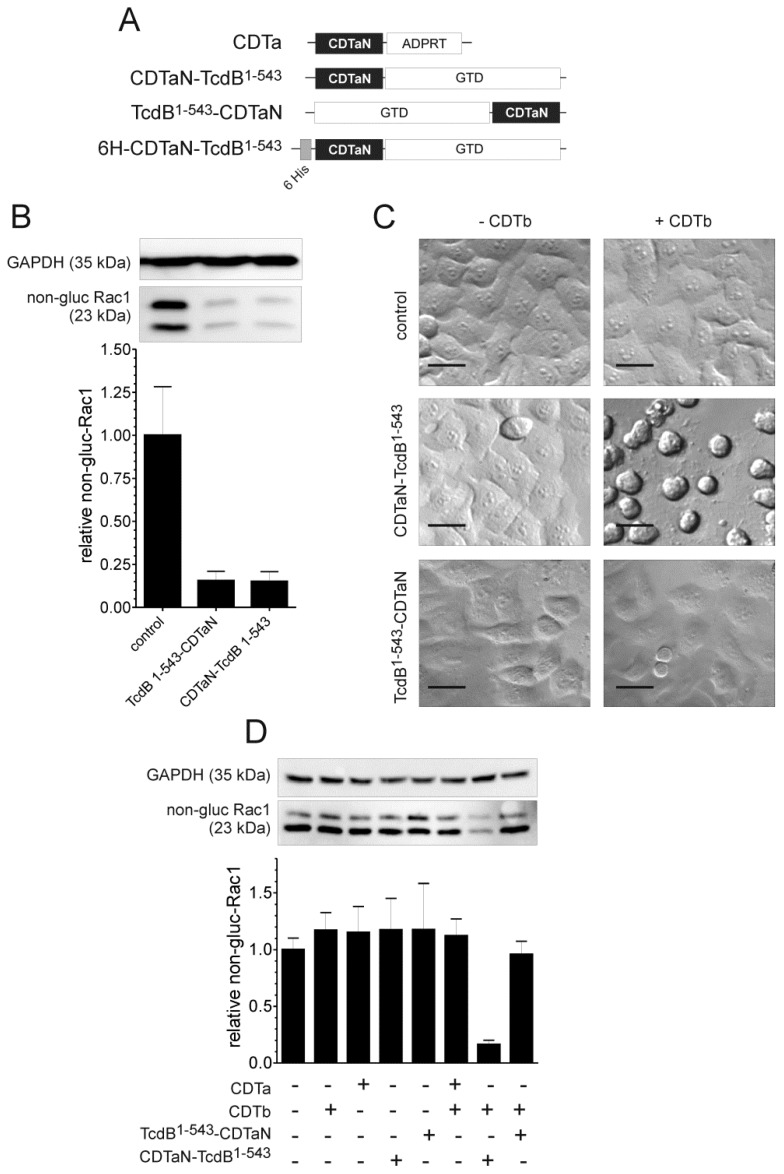
Validation of CDTb/CDTaN as a delivery system for the glucosyltransferase domain of TcdB. (**A**) CDTaN constructs used in this study; (**B**) *In vitro* glucosyltransferase activity of TcdB^1–543^–CDTaN and CDTaN–TcdB^1–543^. Glucosylation was tested in crude HEp-2 cell lysates separated by 12.5% SDS-PAGE and detected by Western blot analysis using specific glucosylation-sensitive Rac1-antibody. Rac1 glucosylation relative to GAPDH as a loading control was quantified, and the data are shown in the bar graph (means ± SD, *n* = 7); (**C**) CDTaN–TcdB^1–540^ but not TcdB^1–543^–CDTaN (each 500 ng/mL) induced typical cell rounding of HEp-2 cells only when delivered by CDTb (1 µg/mL). Scale bars represent 20 µm; (**D**) The bar chart shows the relative amount of non-glucosylated Rac1 in HEp-2 cells treated with the indicated proteins. Representative immunoblots of non-glucosylated Rac1 and GAPDH show the corresponding bands for evaluation. In contrast to the *C*-terminal fusion protein (CDTaN–TcdB^1–543^), the *N*-terminally fused glucosyltransferase (TcdB^1–540^–CDTaN) was not delivered into the cells. Shown are mean values ± standard deviation; *n* = 3–6.

After successful characterization of CDT, we started to genetically engineer fusion proteins of the CDTa adaptor domain (CDTaN) which comprises the amino acids 1–226 of the mature protein. First, we fused the glucosyltransferase domain of *C. difficile* TcdB (amino acids 1–543) to CDTaN. Two different constructs were made with the glucosyltransferase domain (GTD) fused either *C*-terminally to CDTaN (CDTaN-TcdB^1–543^) or *N*-terminally (TcdB^1–543^-CDTaN) (Figure 2A). 

Both constructs were first tested for their glucosyltransferase activity in an *in vitro* glucosylation assay. Figure 2B shows a representative immunoblot of HEp-2 cell lysates incubated with CDTaN–TcdB^1–543^ as well as with TcdB^1–543^–CDTaN that was probed with the glucosylation-sensitive anti-Rac1 antibody (Transduction Laboratories, clone 102) [25]. In cell lysates incubated with either fusion protein, a TcdB^1–543^-catalyzed glucosylation of the substrate GTPase Rac1 was observed. Non-glucosylated Rac1 was decreased to about 80% compared to untreated cell lysate. GAPDH was used as a reference for protein loading. The *C*- and *N*-terminal fusion proteins were subsequently tested in cell culture assays to investigate the intracellular delivery of these toxin domains by the CDTaN/b delivery system. Since glucosylation of Rho-family GTPases induces complete cell rounding, these morphological changes served as the end point for a first screening. The micrographs shown in Figure 2C clearly evidenced that the CDTaN/b system successfully delivered the 60 kDa sized glucosyltransferase domain of TcdB into the cytosol of HEp-2 cells. This was, however, the case only if the GTD was fused *C*-terminally to CDTaN. The opposite construct TcdB^1–543^-CDTaN was not introduced into the target cells, as shown by the unchanged control morphology. Cells treated with only the CDTaN fusion proteins were also unaffected, thereby validating a CDTb-dependent delivery. The intracellular action of TcdB^1–543^ was additionally and specifically tested by probing cell lysates with glucosylation-sensitive anti-Rac1 (Figure 2D). Only in cells treated with CDTaN–TcdB^1–543^ in the presence of CDTb, a decreased level of non-glucosylated Rac1 was observed.

### 2.3. Application of the CDT Delivery System with a Variety of Glucosyltransferase Domains

**Figure 3:**
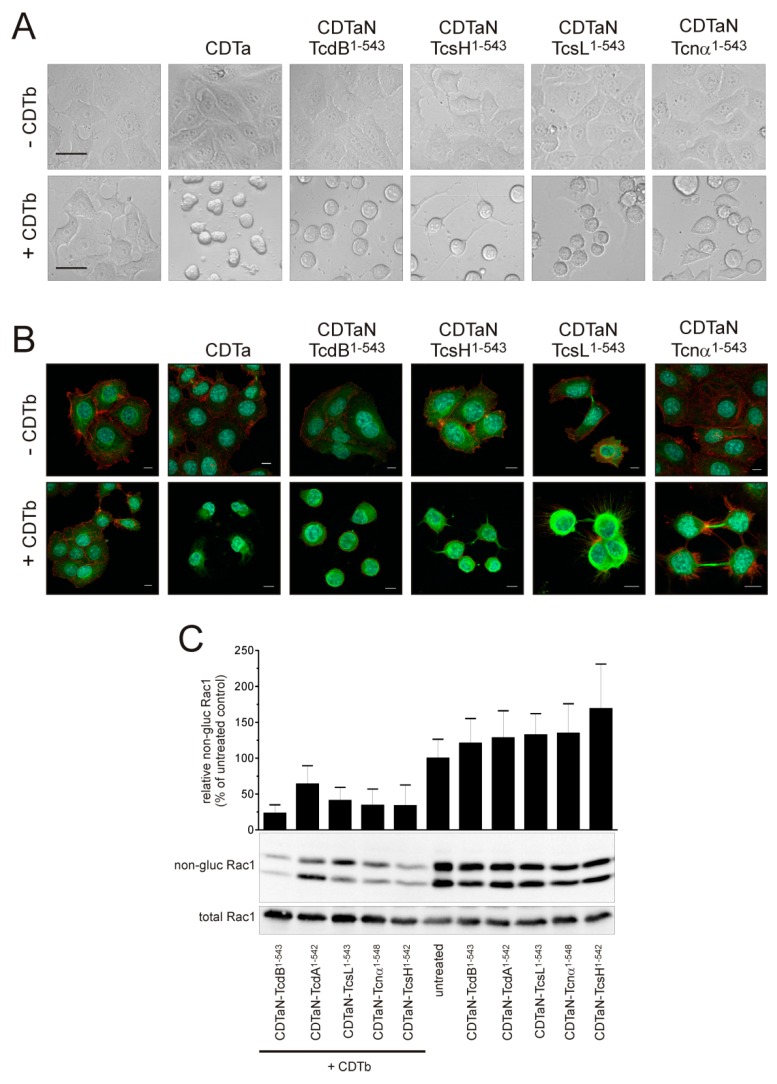
Exploiting the CDT system for standardized delivery of various clostridial GTDs. (**A**) Phase-contrast microscopy of cell rounding assays showing intracellular delivery of CDTa and of all tested CDTaN fusion GTDs (CDTaN–TcdB^1–543^, CDTaN–TcsH^1–542^, CDTaN–TcsL^1–543^, CDTaN–TcnA^1–548^). Scale bars in untreated controls represent 20 µm; (**B**) Immunofluorescence staining of filamentous actin (red), tubulin (green), and the nucleus (blue) of cells treated with the indicated fusion toxins in the absence or presence of CDTb. The scale bars represent 10 µm; (**C**) Indirect proof of Rac1 glucosylation by immunoblot against non-glucosylated Rac1 verifies the intracellular modification of the common substrate GTPase by all fusion toxins.

To simplify the purification of CDTaN–TcdB^1–543^, we cloned the gene into the pQE30 vector, obtaining the 6-His–CDTaN–TcdB^1–543^ fusion protein (see Figure 2A). The *N*-terminal 6-His tag had no effect on the function of CDTaN–TcdB^1–543^ (Figure 3A). This positive result encouraged us to clone the CDTaN fusion proteins of a variety of GTDs from different large clostridial glucosyltransferases. Figure 3A illustrates that not only the GTD of TcdB, but also other GTDs, namely, those of *C. sordellii*, TcsH and TcsL, and of *C. novyi*, TcnA, were successfully delivered into cells. Phase-contrast micrographs showed typical cell rounding when the cells were treated with CDT, with all tested fusion toxins (Figure 3A). Immunofluorescence staining of the actin and tubulin cytoskeleton was performed for a more detailed analysis. In all cases, the actin cytoskeleton was affected, and only remnants of filamentous actin were detected (Figure 3B). In CDTb/CDTaN–TcnA-treated cells, retraction fibers with prominent actin filaments were observed, most probably due to a delayed effect induced by this fusion toxin compared to others. 

In our set of fusion toxins, we found CDTaN–TcdA to be rather instable, with >90% of degraded protein (Supplementary Figure S1). Although CDTaN–TcdA showed transient cell rounding, we did not include this toxin in Figure 3A,B. Instead, morphological changes are shown in supplemental Figure S1. Rac1 glucosylation for all tested glucosyltransferases delivered by the CDTaN/b system is shown in Figure 3C. Rac1 is the common substrate for all tested large clostridial glucosyltransferases [26], and the amount of non-glucosylated Rac1 was only reduced when the fusion toxins were delivered by CDTb. The Rac1 bands detected by glucosylation-sensitive anti-Rac1 antibody (clone 102, Transduction Laboratories) account for Rac1 and truncated Rac, respectively, in HEp-2 cells and were glucosylated as well by the toxins. The upper band was also detected by an anti-Rac1 antibody (clone 23A8, Upstate).

### 2.4. Cell Surface Binding of CDTaN Fusion Proteins 

**Figure 4:**
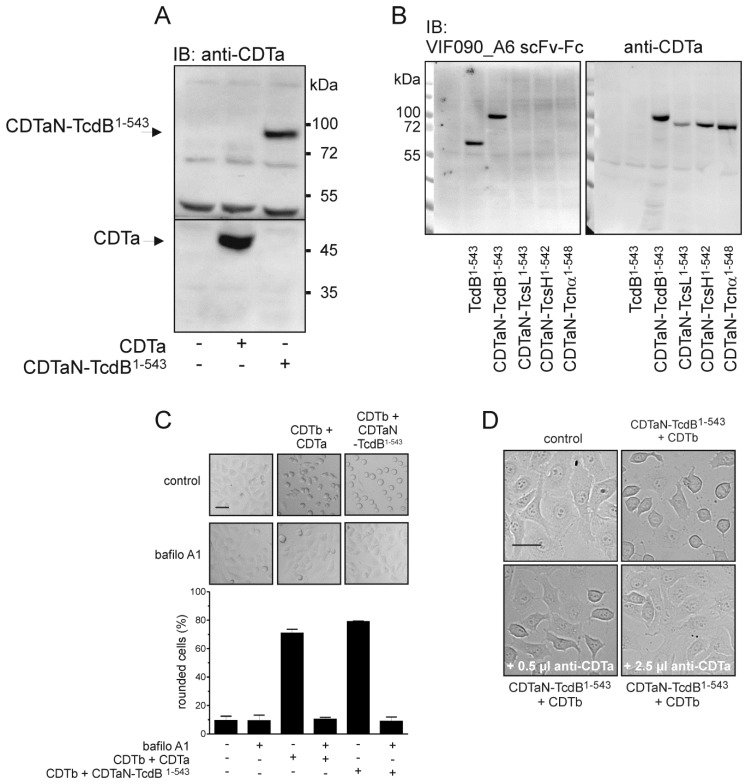
Detection and neutralization of CDTaN fusion toxins. (**A**) Binding of CDTa and CDTaN–TcdB^1–543^ to the outer cell surface detected by immunoblot analysis using anti-CDTa rabbit serum; (**B**) Binding of all CDTaN fusion toxins and of TcdB^1–543^. Shown is the detection by TcdB^1–540^-specific scFv-Fc VIF090_A6 (left panel) and by anti-CDTa rabbit serum (right panel); (**C**) Inhibition of pH-dependent delivery of CDTa and CDTaN–TcdB^1–543^ by the v-ATPase inhibitor bafilomycin A1; (**D**) Neutralization of CDTaN–TcdB^1–543^ by anti-CDTa rabbit serum within the cell culture assay. Scale bars in controls C and D represent 20 µm.

To further characterize the application of fusion toxins, we investigated cell surface binding and inhibition of uptake. Since we previously detected cell surface binding of the isolated GTD of TcdB [27], we also tested the binding of CDTaN–TcdB^1–543^ to the outer cell surface in the absence of CDTb. Immunoblot analysis showed binding of CDTaN–TcdB^1–543^ as well as of CDTa to the cells even in the absence of CDTb (Figure 4A). 

Binding of CDTaN was validated by detection of all used CDTaN fusion toxins (Figure 4B). The meaning of extracellular binding of CDTa is discussed later. Nevertheless, for uptake into the cytosol, CDTb is mandatory. The translocation of CDTaN fusion proteins was induced by a pH shift in endosomes, as it could be inhibited by the v-ATPase inhibitor bafilomycin A1 (Figure 4C). Here, we also tested the neutralizing effect of our polyclonal rabbit serum raised against CDTa. The serum completely inhibited cell rounding induced by CDTaN–TcdB^1–543^ over the observed time period of 6 hours (Figure 4D).

## 3. Discussion

**Figure 5:**
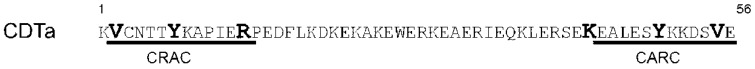
Does CDTa possess a functional cholesterol binding site? A cholesterol recognition amino acid consensus (CRAC) motif (L/V–X_(1–5)_–Y/F/W–X_(1–5)_–K/R) and the reversed CARC motif (K/R–X_(1–5)_–Y/F/W–X_(1–5)_–L/V) can be found in the *N*-terminal 55-amino acid sequence of CDTa.

In this study, we evaluated the *C. difficile* binary toxin CDT as a valuable cell biological tool for intracellular delivery of proteins. We briefly characterized our recombinant CDTa and CDTb with respect to ADP-ribosyltransferase activity *in vitro* and in cell culture assays. After validating the recombinant CDT system, we adapted the CDTaN/b delivery system for intracellular delivery of glucosyltransferase domains from different bacterial pathogenicity factors. Neither the description of CDT nor the use of binary toxins as Trojan horses for delivery of proteins into cells is new. The novelty of this study is that CDT was successfully adapted for protein delivery for the first time, demonstrating to be equivalent to the *B. anthracis* PA system and the *C. botulinum* C2 system. We have shown that not only ADPRTs, but also completely different enzymatically active proteins or protein domains can be delivered into cells by binary toxins. This was reported before only in a single study using the PA–LFn system [17]. Although CDTb has only 28% and 38% sequence identity with PA or C2II, respectively [28], we here present the Iota-like CDTaN/b system as equivalent to the other binary delivery systems. Several reports showed the employment of binary toxins for protein delivery, as summarized by Barth and Stiles [28]. A closer look reveals that most of all bacterial ADPRTs, especially exoenzymes C3^bot^ from *C. botulinum* and C3^lim^ from *C. limosum* but also SpvB from *Salmonella enterica*, were successfully delivered into cells [18,20,29,30,31]. The C2I–C3 fusion proteins have been tested primarily on macrophages. The exoenzyme C3 fusion proteins, however, have the disadvantage of entering some cells by two ways: by C3-specific uptake or by specific C2II-mediated uptake. The exoenzymes C3^bot^ or C3^lim^ can enter macrophages without further transporting devices but enter these cells more efficiently by exploiting the C2I/C2II delivery system [32]. Other reports clearly showed the uptake of isolated C3^bot^ not only into macrophages but also into a variety of cells, such as murine hippocampal HT22 cells, stem cell-derived neurons from mice, SH-SY5Y cells, or even Chinese hamster ovary cells (CHO) by using vimentin as a binding structure [33,34,35,36]. Thus, by applying C2I–C3 fusion proteins, it is hard to differentiate between specific C3 uptake and C2II-mediated uptake into cells. Our system clearly validated a CDTb-dependent uptake of non-ADPRT fusion toxins. In our studies, we experienced that CDTa and the fusion proteins of CDTaN bound to the cell surface of the target cells independently of CDTb. This was shown by specific immunoblots against CDTa or TcdB^1–543^. Binding to cells did not result in the translocation and intracellular action of the fusion toxins, which was only observed in the presence of CDTb. The binding of CDTa to the cell surface is interesting for two reasons: (1) confocal microscopy can hardly differentiate between the extra- and intra-cellular leaflets of vesicle membranes, making functional assays for translocation inevitable to prove membrane translocation; (2) If CDTa is membrane-associated, this might important for the intracellular localization of cargoes. Further investigations have to show the intracellular localization and distribution to subcellular compartments of binary fusion toxins. An eventual intracellular membrane association of proteins clearly limits the range of application for this kind of carrier. Yet, we do not know the nature of the cell surface interaction of CDTa. According to the amino acid sequence, it can be hypothesized that CDTa binds cholesterol with its *N*-terminal part. Fantini and coworkers described a mirror code of the cholesterol recognition amino acid consensus (CRAC) sequence (Figure 5) [37]. 

Although the CRAC motif and the reversed CARC motif are separated by 31 amino acids in CDTa, which is more than it can be found in reference membrane-associated proteins shown by Fantini and coworkers, we cannot exclude cholesterol binding for CDTa. Unfortunately, the 3D structure of CDTa does not contain the first CRAC motif, giving no information about the vicinity of the CRAC and CARC motifs (PDB code 2WN4). 

Our present data reveal that CDTaN is not able to deliver *N*-terminally fused cargo into cells, as it was known for iota toxin [23]. This is most probably due to impaired transition through the pore built by CDTb oligomers. The small *N*-terminal 6×His tag, however, does not hamper the function of CDTa. The non-functionality of *N*-terminal fused proteins is a disadvantage of the CDT system, excluding cargoes, where a free *N*-terminus is required. In a set of experiments, we extended the application of CDTaN fusion proteins for a trial study of standardized delivery of homologous protein domains. As shown in Figure 3, CDTaN fusion proteins containing the pathogenic domains of widely known large clostridial glucosyltransferases were introduced into cells under standardized conditions. The translocation of CDTa or CDTaN fusion proteins occurred under acidic conditions, and could be inhibited by bafilomycin A1. The typical morphotypes of cells treated with CDTaN fusion GTDs corresponded with that known to be induced by full-length glucosyltransferases. This was demonstrated by the appearance of the “*sordellii*-like” clustering morphotype which was also induced by CDTaN–TcsL^1–543^ and the patchy cell rounding induced by TcnA. Our experiments thus validated the CDT system as a protein delivery system for pathogenic toxin domains, enzymes, or effector proteins that do not possess autotransporter characteristics. A variety of effector proteins from pathogenic bacteria that have glucosyltransferase activity, such as *Legionella pneumoniae* SetA or Lgt1as well as the effector protein CT166 of *Chlamydia trachomatis* [38,39,40], were characterized by transfection experiments. Our system offers an alternative approach to investigate the direct effects of such effector proteins in target cells. 

A further putative application, especially of the CDT homologous iota toxin from *C. perfringens*, is the use in anti-cancer therapy. It is known that the lipolysis-stimulated lipoprotein receptor (LSR) is overexpressed in cancer cells [41]. Iota toxin as well as CDT use this receptor to enter eukaryotic cells. This makes binary toxins using LSR as a receptor an attractive tool to preferentially target cancer cells, as previously described for iota toxin [42,43]. On the other hand, a clear disadvantage of the CDT or iota system is that these toxins cannot be applied to cells that lack LSR without transduction of the respective receptor [13,42]. The binary toxin systems additionally bear the advantage of possible retargeting to cell-specific receptors by exchange of the receptor binding domain of the B subunits of toxins. Likewise, the LF/PA system from *B. anthracis* was used in combination with the pathogenic domain of TpeL to target oncogenic Ras [44]. These applications illustrate even a pharmacological potential of binary toxins.

In summary, our study encourages the further use of the CDTaN/b system for delivery of a variety of proteins, including antibody fragments (intrabodies). More research has to be done to evaluate the intracellular localization and the nature of cargoes that can be delivered. Although the GTDs appear to have a significant size of 60 kDa, these protein domains do not possess disulfide bonds, making membrane translocation with accompanied pore transition more probable compared to proteins with a more rigid tertiary structure. Thus, our data contribute to the validation of binary toxins as valuable tools in cell biology.

## 4. Materials and Methods 

### 4.1. Cloning of CDTa, CDTb, and CDTaN Fusion Proteins

The complete ORFs of CDTa and CDTb were amplified by PCR from genomic DNA of *C. difficile* strain R20291 (GenBank: FN545816.1). Since both genes code for a 42-amino acid (126 base pair)-long leader sequence for secretion of proteins, we only cloned the sequences bp 127–389 for CDTa and bp 127–2628 for CDTb into the pGEX-2T vector to genetically engineer the GST fusion proteins of the mature CDTa and CDTb. CDTaN was also ligated into the pQE30 vector for generation of *N*-terminally 6xHis-tagged CDTaN fusion proteins. Table 1 lists the primers that were used for cloning. 

### 4.2. Purification and Activation of CDTa and CDTb

GST–CDTa, GST–CDTaN–TcdB^1–543^ and GST–CDTb were expressed in *Escherichia coli* following a standard protocol. Gene expression was induced by 100 µM isopropyl-*β*-d-thiogalactopyranosid when the bacterial cultures reached an OD_600_ of 0.6. The GST fusion proteins were affinity purified via glutathione-sepharose (GE Healthcare, Dornstadt, Germany) by gravity flow, and the proteins of interest were released either by thrombin (0.06U/µg protein, 4 °C overnight for CDTa and CDTaN–TcdB^1–543^) or by elution with 10 mM glutathione (CDTb). Eluted GST–CDTb was directly activated by trypsin (0.2 µg/µg protein, 30 min at room temperature). Trypsin was inactivated by 2 mM 4-(2-Aminoethyl)benzensulfonylfluorid, and the solution was dialyzed against PBS overnight. The 6×His-tagged proteins were purified using Protino Ni-IDA columns (Macherey-Nagel, Düren, Germany) following a standard protocol supplied by the manufacturer. The elution buffer was exchanged via ZEBA desalting columns (Thermofisher, Bonn, Germany), and the proteins were stored in 10 mM Tris-HCl, ph 7.4, 20 mM NaCl at −80 °C. In the following, CDTa always means the mature protein without the leader sequence, and CDTb always stands for trypsin-activated CDTb if not stated otherwise, since it was only used for cell culture experiments to deliver CDTa and CDTaN fusion proteins into target cells.

### 4.3. In Vitro ADP-Ribosylation and Glucosylation Assays

ADP-ribosylation was performed with rabbit muscle actin (1 µg) or HEp-2 crude cell lysates (100 µg) in 0.1 mL ribosylation buffer (20 mM HEPES, pH 7.4, 100 mM NaCl_2_, and 100 µM NAD). For [^32^P]ADP-ribosylation, the buffer was additionally supplemented with 0.5 µCi [^32^P]NAD. The reaction was started by addition of 200 ng CDTa per sample or as indicated and incubation for 45 min at 37 °C. The reaction was stopped by addition of 5-fold Laemmli buffer and heating at 95 °C for 3 min. The samples were resolved by SDS-PAGE. Radioactive [^32^P]ADP-ribosylation was detected by filmless autoradiography (Canberra Packard) of Coomassie-stained gels or by gel shift assay and specific immunoblots in case of non-radioactive ADP-ribosylation. In vitro glucosylation of Rac1 was performed by harvesting HEp-2 cells in glucosylation buffer (100 mM KCl, 1 mM UDP-glucose, 1 mM MgCl_2_). The cells were homogenized by sonification, and 100 µL, containing 100 µg of proteins, was used for glucosylation. To start the reaction, CDTaN–TcdB^1–543^ or TcdB^1–543^–CDTaN (300 ng each) were added to the cell homogenates, and the reaction was incubated at 37 °C for 1 h. The reaction was stopped by addition of Laemmli buffer and heating at 95 °C for 5 min, and Western blot with glucosylation sensitive Rac1- antibody was performed.

### 4.4. Cell Culture and Cell Rounding Assay 

HEp-2 cells were kept in Minimum Essential Medium (MEM) Eagle supplemented with 10% fetal bovine serum, 100 μM penicillin, and 100 μg/mL streptomycin in a humidified atmosphere of 5% CO_2_ at 37 °C and passaged three times a week. For the experiments, cells were seeded in a defined volume to reach a confluency of about 70% on the day of the experiment. For the cytotoxicity assays, the cells were incubated with either 500 ng/mL of the CDTa or CDTaN constructs, 1000 ng/mL of CDTb, or the combination of both at 37 °C for 4 h, if not stated otherwise. For the bafilomycin experiments, the cells were pre-incubated with a concentration of 500 nM bafilomycin A1 (Calbiochem/Merck, Darmstadt, Germany). In the case of the neutralization assays, 0.5 µL or 2.5 µL of CDTa-specific polyclonal rabbit serum was pre-incubated with 500 ng/mL of CDTaN–TcdB^1­–543^ at 37 °C for 15 min and added to the cells together with 1000 ng/mL of CDTb, followed by an incubation period at 37 °C for 2 h. The morphological changes were documented by an Axiovert 200 microscope (Carl Zeiss, Feldbach, Austria). To investigate the status of glucosylated Rac1, the cells were seeded in 3.5 cm cell culture dishes and harvested in 100 µL Laemmli buffer after treatment with the CDTa/CDTaN constructs and/or CDTb. After sonification for 3 s, the samples were incubated at 95 °C for 5 min and subjected to SDS-PAGE and Western Blot.

### 4.5. Toxin Binding Assay 

The extracellular binding of the CDTa and CDTaN fusion proteins to HEp-2 cells was investigated by Western blot. The cells in 24-well plates were incubated with 2 µg/mL of CDTa and CDTa fusion proteins in culture medium for 30 min at 4 °C. Afterwards, the cells were rinsed twice with PBS and scraped off from the cell culture wells in 100 µL of Laemmli buffer. The samples were briefly sonicated for lysis, heated at 95 °C, and subjected to SDS-PAGE and immunoblot.

### 4.6. Immunostaining and Fluorescence Microscopy

For immunostaining, HEp-2 cells were cultured in µ-Slide eight-well cell culture chambers (ibidi GmbH, Planegg, Germany). Five hours after toxin treatment, the samples were fixed with 4% PFA in PBS at 4 °C for 10 min and directly used for staining. The fixed cells were blocked and permeabilized in PBS supplemented with 5% milk powder and 0.3% Triton X-100 for 10 min at RT. After blocking, the cells were incubated at 4 °C overnight in the primary antibody solution: 1 µg/mL mouse anti-tubulin antibody B-5-1-2 (Sigma-Aldrich, Hamburg, Germany) in 1% milk powder-supplemented PBS (MPBS). The day after, the cells were incubated in goat anti-mouse IgG Alexa 488 (A11001 Invitrogen/Thermo Fisher Scientific, Darmstadt, Germany) secondary antibody solution for 1 h at RT in the dark. F-actin staining was performed with MPBS supplemented with Phalloidin-iFluor 555 (AAT Bioquest, Sunnyvale, CA, USA), while counterstaining was carried out by incubating the samples with 100 ng/mL DAPI (Thermo Fisher Scientific, Darmstadt, Germany) in PBS for 10 min. At the end of every described step, the samples were washed three times with PBS for at least 5 min. 

The stained cells were imaged by a confocal laser scanning microscope (SP8, Leica Microsystems). All the images were taken using constant acquisition parameters and were equally processed with Leica Application Suite X (Leica Microsystems) and Fiji [45].

### 4.7. Generation of a Monoclonal Anti-TcdB Antibody

Antibodies against TcdB were selected in the scFv-format from the human naive antibody gene libraries HAL9/10 [46]. The selection and screening were performed as described before [47]. In brief, for antibody selection, the scFv phages were incubated on TcdB^1–1852^, immobilized on Costar High-Binding microtiter plates (Sigma-Aldrich Chemie GmbH, Munich, Germany). After three rounds of panning, 94 clones were screened for the production of TcdB^1–1852^-binding scFvs by antigen ELISA. After the identification of the scFv producing clones, plasmid DNA was isolated, and the antibody DNA was sequenced. The scFv was recloned into pCSE2.6-hIgG1-Fc-XP using NcoI/NotI for mammalian production as scFv-Fc, an IgG-like antibody format (VIF090_A6). The production and purification were performed as described before [48].

### 4.8. Polyclonal Anti-CDTa Rabbit Serum

To obtain CDTa-specific polyclonal rabbit serum, a female New Zealand rabbit was immunized with 100 µg purified recombinant CDTa in PBS. A boost was made 4 and 6 weeks after immunization, again with 100 µg antigen each. Eight weeks after the first immunization, blood was collected, and the anti-CDTa serum was tested for specificity. All animal treatments were performed according to the national Protection of Animals Act (Permission No. 33-42502-03A351) at our central Laboratory Animal Science.

### 4.9. Western Blot

Proteins resolved by SDS-PAGE were transferred onto nitrocellulose by semi-dry blotting. The nitrocellulose was blocked with 5% [*w/v*] non-fat dry milk powder in TBS-Tween (20 mM Tris, pH 7.4, 50 mM NaCl, 0.02% [*v/v*] Tween-20) for 30 min. Specific first antibodies (1 µg/mL) were applied in TBS-T over night at 4 °C. After washing with TBS-T, appropriate horseradish-conjugated secondary antibodies were applied for 45 min. The nitrocellulose was washed three times with TBS-T, and the detection of the bound antibodies was done by a chemiluminescence reaction (Supersignal West femto, Pierce, Germany).

## Figures and Tables

**Table 1:** Primers used for cloning of the CDTa and CDTb constructs.

	Name	Base Sequence (3’-5’)
pGEX-2T constructs	CDTa (aa 1–463) s	TAGGATCCAAAAAATTTAGGAAACATAAAAGGATTAG
	CDTa (aa 44–463) s	TTAGGATCCGTTTGCAATACTACTTACAAGGC
	CDTa (aa 1–463) a	ATGAATTCTTAAGGTATCAATGTTGCATCAAC
	CDTb (aa 1–876) s	TAAGATCTAAAATACAAATGAGGAATAAAAAGGTATTAAG
	CDTb (aa 43–876) s	TAAGATCTGAAATTGTAAATGAAGATATACTCCC
	CDTb (aa 1–876) a	ATGAATTCCTAATCAACACTAAGAACTAATAACTCTC
pQE30 constructs	CDTaN Bam s	AATGGATCCGTTTGCAATACTACTTACAAGG
	CDTaN Kpn a	AATGGTACCATCATCTTTAAAATCAAGACTATTTAC
	GTD TcdB Kpn s	AATGGTACCATGAGTTTAGTTAATAGAAAACAGTTAG
	GTD TcdB Hind a	AATAAGCTTTTAAAGAGAACCTTCAAAATAATTCCTTTTATATTC
	GTD TcdA Kpn s	AATGGTACCATGTCTTTAATATCTAAAGAAGAG
	GTD TcdA Hind a	AATAABCTTTTAAAGAGATCCACCAGTATAATCTC
	GTD TcnA Kpn s	AATGGTACCATGCTTATAACAAGAGAACAATTAATG
	GTD TcnA Hind a	AATAAGCTTTTAGAGAGTTCTTCCTATATAAGTTTTTATC
	GTD TcsH Kpn s	AATGGTACCATGTCTTTAATATCTAAAGATGAATTAATAAAAC
	GTD TcsH Hind a	AATAAGCTTTTAAAGAGATTCCTGAGTATAATCTCTTAC
	GTD TcsL Kpn s	AATGGTACCATGAACTTAGTTAACAAAGCCC
	GTD TcsL Hind a	AATAAGCTTTTAAAGTGCACCTTCAAAATAACC

## Supplementary Materials

The following are available online at http://www.mdpi.com/2072-6651/10/6/225/s1, Figure S1: Instable CDTaN-TcdA^1–542^ fusion protein.
