# Supplementary material for: The Binary Toxin CDT of Clostridium difficile as a Tool for Intracellular Delivery of Bacterial Glucosyltransferase Domains
Source: Toxins (Basel). 2018 Jun 1;10(6):225. doi: 10.3390/toxins10060225 (PMC6024811; doi:10.3390/toxins10060225)
Supplement: Supplementary file 1 [file toxins-10-00225-s001.pdf]

Supplementary Materials: The Binary Toxin CDT of *Clostridium difficile* as a Tool for Intracellular Delivery of Bacterial Glucosyltransferase Domains

Lara-Antonia Beer, Helma Tatge, Carmen Schneider, Maximilian Ruschig, Michael Hust, Jessica Barton, Stefan Thiemann, Viola Führner, Giulio Russo and Ralf Gerhard

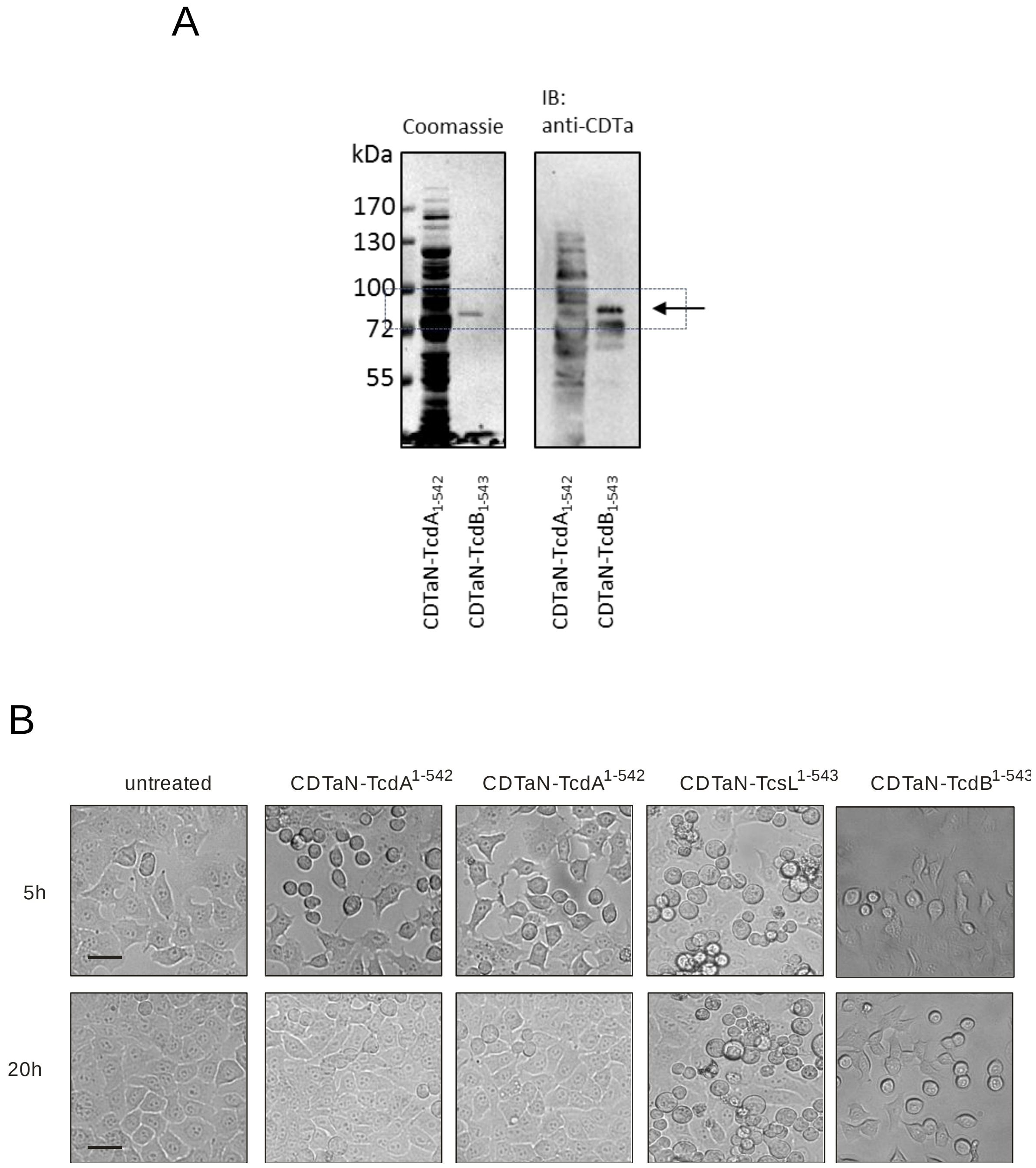

Figure S1. Instable CDTaN-TcdA$^{1-542}$ fusion protein. **A**) Eluate from Ni^{2+}-affinity purification of 6xHis tagged CDTaN-TcdA$^{1-542}$ (91 kDa) by Ni-Ida columns (Macherey-Nagel) showed enrichment of unspecific proteins in Coomassie-stained 7.5% SDS-PAGE (left panel). For comparison, purified 6xHis tagged CDTaN-TcdB$^{1-543}$ (91 kDa, arrow) is shown. Half of the SDS-gel was blotted onto nitrocellulose and specific detection of the same CDTaN-fusion proteins was performed by immunoblot analysis

with anti-CDTa polyclonal rabbit serum (right panel). CDTaN-TcdB$^{1–543}$ along with few degradation products, that were not visible in Coomassie gel, were specifically detected. On the contrary, no specific band accounting for CDTaN-TcdA$^{1–542}$ was observed. Thus, 6xHis tagged CDTaN-TcdA$^{1–542}$ is below detection limit in SDS-PAGE and Immunoblot analysis. **B**) For a sensitive proof of functional CDTaN-TcdA$^{1–542}$ a cell rounding assay with HEp-2 cells was performed. Cells were seeded in a 24-well plate at a density of approximately 5 × 105 cells/well in Minimum Essential Medium Eagle supplemented with 10% fetal bovine serum, 100 µM penicillin and 100 µg/ml streptomycin and were let grown over night. 1000 ng/ml activated CDTb in combination with 500 ng CDTaN-fusion protein (as indicated) were applied. In case of CDTaN-TcdA$^{1–542}$ with unknown protein concentration, 2 µl of protein solution was added to 1 ml cell culture medium containing 1000 µg activated CDTb. Morphological changes of cells were monitored by phase contrast microscopy 5 h and 20 h after addition of toxins. Transient morphological changes were induced by CDTaN-TcdA$^{1–542}$ in some cells (shown are two replicates) after 5 h. After 20 h of incubation, no morphological changes of cells were observed anymore. In contrast, HEp-2 cells were affected by CDTaN-TcdB1-543 or CDTaN-TcsL$^{1–543}$ after 5 h, and the effect was observed even after 20 h of incubation. Scale bar in untreated controls represent 25 µm.
